# Factors associated with suicidality among school attending adolescents in morocco

**DOI:** 10.3389/fpsyt.2022.885258

**Published:** 2022-08-08

**Authors:** Abdallah Tom, Ziyad R. Mahfoud

**Affiliations:** ^1^Department of Medical Education, Weill Cornell Medicine-Qatar, Doha, Qatar; ^2^Division of Epidemiology, Department of Population Health Sciences, Weill Cornell Medicine, New York, NY, United States

**Keywords:** suicide, Suicidality, adolescents, Morocco, MENA, wellbeing

## Abstract

Suicide amongst adolescents is a growing epidemic accounting for 6% of all adolescent deaths. Even though 79% of adolescent suicides occur in low- and middle-income countries, where suicide is the second leading cause of death, research is relatively lacking. As such, we aim to gain a greater understanding of suicide in said countries by assessing ideation and planning and associated factors in Morocco. Global Schools Health Survey data was analyzed. Approximately 14.4 and 12.9% reported ideation and planning respectively during the prior year in 2016, indicating a decrease from the reported rates of ideation of 16.0 and 17.0% and planning of 14.6 and 15.0% in 2006 and 2010 surveys respectively. Increased ideation was found to be positively associated with identifying as female and increasing age, whereas planning was positively associated with a lower educational level and living in a rural area. Both were positively associated with increased hunger frequency. Several factors increased the likelihood of ideation: bullying, feeling lonely, current cigarettes smoking, and current marijuana use. Studying factors associated with suicide is challenging, alternatively, factors affecting ideation and planning can be assessed. Sociocultural differences may impact trends in a specific region, though countries in said region may have comparable trends. The study adds to the limited data available in the region. Reverse causality and under-reporting could be the main limitations of this study. Interventions taking into account those results should be tested to decrease such a prevalence.

## Introduction

Suicide among adolescents is a growing epidemic affecting youth globally accounting for 8.5% of all causes of mortality amongst young adults aged 15–29; it is the second cause of death among adolescent worldwide (1). Even though the absolute number of suicide cases among adolescents is lower than that of older adults, it poses a significant public health threat affecting individuals, families, and communities alike through multiple dimensions including economic, social and psychological (2). Although low- and middle-income countries account for 79% of suicide cases among adolescents, research delving into suicide, associated risk factors, and prevention is often neglected in said countries (3). Prior studies have highlighted the scarcity of data and research in Muslim-majority countries, which in turn impacts assessment of prevalence, effective intervention planning and education, and prevention (4). Suicide is defined as the act of taking one's own life intentionally. While suicidal ideation is defined as having thoughts of wishing you were dead (would be better off not living) but without having plans to commit suicide, and suicidal planning is having a detailed suicidal plan (5). Though the rates of suicidal ideation and suicidal planning are higher than suicidal attempts and completed suicide, the prevalence of completed suicide is staggering. According to the National Institute of Mental Health, in 2018 1.4 million Americans attempted suicide and 47,173 died as result of suicide, of which 6,769 were between the ages of 10 and 24 (6). According to the WHO, every 40 seconds one person dies as a result of suicide.

Suicide is complex to understand for both the victims and researchers alike. As such, it is a multidimensional complex public health threat that is challenging to truly decipher and understand. Although suicidality is hard to predict, there has been multiple studies exploring the potential risk factors of suicide including psychological, sociological, and biological factors. A study exploring suicide and its associated risk factors on both population and individual level concluded that individual risk factors include family history, loneliness, traumatic events, interpersonal stressors, and non-mental chronic disease (7). About 20% of adolescents experience mental health disorders, depression and anxiety being most common, which precipitate most of suicide and suicidal attempts (8, 9). It is hypothesized that adolescents would utilize online search engines to explore methods for suicide, which could be used to explore associations and target interventions. A study conducted in Italy, found an association between Google search volumes for the term “suicide” and the volume of death due to suicide in the following 3 months; however, no correlation was found with the terms “how to commit suicide” and “commit suicide.” The difference in correlation is thought to be due to the search being linked to other factors such as personal interest and suicide bereavement (10). According to the World Health Organization and the United Nations Children's Fund, health systems and international organizations need to place more emphasis on the importance of adolescents' mental health (1).

Suicide is associated with a wide variety of risk factors and demographics that are different/heterogeneous between different regions of the world (1). Research studies conducted in Europe and North America indicate that the rate of suicide differs between genders and a correlation exists with multiple associated factors including life satisfaction and mental illness (11). Some studies have shown that the male sex, parental and/or personal mental health problems, belonging to the LGBTQI+ community, substance intoxication, substance use disorders, and pathologic internet use are associated with increased risk of suicide (12). The rates of completed suicide amongst boys is 3 times that of girls; however, the rates of incomplete suicidal attempts are 2 times higher in girls as compared to boys. It is estimated that between 1 in 50 to 1 in 100 suicidal attempts are completed suicides. The gender discrepancy in the rates of completed suicide and suicidal attempts is hypothesized to be due to girls using less lethal methods as compared to boys (12). However, relatively limited research is conducted in the Middle East and North Africa due to multiple variables including lack of reporting due to the associated social stigma and cultural restrictions. The available studies often combine countries together or use older datasets. Socio-cultural differences play a significant role in the perception of suicide, which may impact reporting of suicide cases. When assessing suicidality and associated risk factors, it is important to assess both suicidal ideation and planning within a specific sociocultural context. Unfortunately, due to limited suicide research in the Eastern Mediterranean Region, there is relatively limited information as compared to other regions (13). A 2017 study assessing suicide in Morocco using the 2010 dataset of the Global Schools Health Survey found that 16.6% of adolescents have expressed suicidal ideation, and a positive correlation existed between suicide and increasing age, food insecurity, anxiety, loneliness, bullying, substance abuse, and cigarette and marijuana smoking (14). Additionally, a recent study evaluating suicidal ideation amongst adolescents in Lebanon found that out of the 1,810 adolescents enrolled, 28.9% expressed suicidal ideation, which was associated with psychological abuse, child physical abuse, alcohol dependence, fear, impulsivity, bullying, internet addiction and identifying as female (15).

The following study primarily aims are to 1) estimate the prevalence of suicidal ideation and suicide planning amongst school attending adolescents in Morocco and compare it to prevalence of other countries during the same period, 2) explore associations between suicidal ideation and planning and demographic variables and 3) explore the potential association between suicidal ideation and planning and risky behaviors amongst school attending adolescents. We hypothesis that suicidality is positively associated with worse mental health wellbeing and increased risky behaviors.

## Materials and methods

### Study setting and sample

Morocco, located in the Southern Mediterranean in Northwest Africa, is an amalgamation of African, Arab, and European cultures 15. According to the world bank, Morocco is classified as a lower middle-income country with a population of approximately 37 million, of which 30% are youth between the ages 15 and 29 (16, 17). Though multiple languages are spoken across Morocco, Arabic is the official national language (18). Over the past two decades, primary school enrollment significantly increased, and it was reported to be 99.1% in 2018 (19).

The following study is a secondary data analysis of an open access data available for the cross-sectional study the “Global Schools Health Survey (GSHS)” conducted in 2016 in Morocco. The GSHS is a collaborative joint effort between the World Health Organization and the United States Centers for Disease Control and Prevention to assist countries globally to accurately assess behaviors amongst school attending young adolescents with low administrative cost. The GSHS is a self-administered questionnaire exploring 10 pillars: Alcohol use, Dietary behaviors, Drug use, Hygiene, Mental health, Physical activity, Protective factors, Sexual behaviors, Tobacco use, and Violence and unintentional injury (20). Inclusion criteria was based on class level and not on age, recruiting participants in grades 7–12. Exclusion criteria include individuals not enrolled in school at the time of the study. Necessary ethical approvals were obtained by the national authorities such as Ministries of Public Health and Education. Participation in the survey is voluntary, and students may opt out.

A total of 6,745 school-attending adolescents between the ages of 13 and 17 participated in the study, with a student response rate of 93% 23. Participants were enrolled in schools in either rural or urban areas in Grades 1 ASC - 2nd yr. Bac (equivalent to grades 7–12).

### Measures

The Global Schools Health Survey that was conducted in Morocco assessed, except for alcohol use and sexual behaviors, all other 8 core modules in the questionnaire.

Suicidality was assessed by measuring suicidal ideation and suicidal planning, using the questions “During the past 12 months, did you ever seriously consider attempting suicide?” and “During the past 12 months, did you make a plan about how you would attempt suicide?” respectively. Participants' demographics including age, sex, weight, educational level, hunger frequency, and school setting were collected. The four questions regarding parental involvement were first dichotomized into yes or no as per the following: “Reported that their parents or guardians most of the time or always checked to see if their homework was done,” “Reported that their parents or guardians most of the time or always understood their problems and worries,” “Reported that their parents or guardians most of the time or always really knew what they were doing with their free time,” and “Reported that their parents or guardians never or rarely went through their things without their approval.” The variable parental involvement was then the sum of the previous four dichotomized variables with higher scores indicating higher levels of parental involvement. Additionally, mental health and wellbeing were assessed using two questions: “Most of the time or always felt lonely” and “Most of the time or always were so worried about something that they could not sleep at night.” Victimization due to bullying was assessed with “Were bullied during the past 30 days.” Additionally, substance use was measured using multiple different questions: “Currently smokes cigarettes,” “Currently uses marijuana,” “Ever used amphetamines or methamphetamines,” and “Used drugs before age 14 years.”

### Statistical analysis

Demographics were summarized using frequency distributions. Chi-squared tests were used to evaluate the association between different demographic variables and suicidal ideation and planning. Univariate and Multivariate logistic regressions were used to explore the simultaneous associations between potential associated factors and suicidal ideation and planning while controlling for age, sex, school grade, weight, parental involvement, and hunger frequency. Unadjusted and Adjusted Odds Ratios and their 95% confidence intervals were reported. A *p*-value less than or equal to 0.05 was considered significant. The analysis was conducted using IBM SPSS Statistics Version 26.0, Armonk NY, USA.

## Results

### Global trends

The prevalence of suicidal ideation and planning amongst adolescents in Morocco in 2016 was 16.0% (95% CI: 15.1–17.0) and 14.6% (95% CI: 13.7–15.5) respectively. During the prior decade the prevalence of both suicidal ideation and planning increased from 14.4 and 12.9% in 2006 to 17.0 and 15.0% in 2010. Using GSHS data conducted around the same year, the results in Morocco were comparable to that of other Eastern Mediterranean Region (EMR) countries such as Lebanon. Liberia had the highest percentage of both suicidal ideation and planning amongst school attending adolescents with 26.8 and 36.5% respectively in comparison to other countries in which the GSHS was administered. Myanmar had the lowest percentage of suicidal ideation and Indonesia had the lowest percentage of suicidal planning: 8.7 and 6.3% respectively. The trends also suggest that the Americas region has the highest overall prevalence of suicidal ideation. Table 1 includes global trends of suicidal ideation and planning from each of the five regions the GHSH was conducted.

**Table 1:** Global trends of suicidal ideation and planning among school attending adolescents based on the GSHS across the 5 different regions.

**Region**	**Country**	**Year of survey**	**Suicidal ideation (%)**	**Suicidal planning (%)**
EMR	Morocco	2016	16.0	14.6
	Morocco	2010	17.0	15.0
	Morocco	2006	14.4	12.9
	Lebanon	2017	17.7	8.9
	Kuwait	2015	17.2	17.2
	Afghanistan	2014	19.1	17.5
	Yemen	2014	16.1	14.4
	Iraq	2012	17.4	16.1
	Tunisia	2008	21.0	13.9
	Jordan	2007	17.8	17.8
Africa	Liberia	2017	26.8	36.5
	Mauritius	2017	15.8	14.3
	Mozambique	2015	18.6	20.6
	Seychelles	2015	21.5	21.8
Americas	Jamaica	2017	26.4	25.0
	Trinidad and Tobago	2017	22.2	21.9
	Anguilla	2016	22.8	22.3
	Guatemala	2015	20.7	16.6
South-East Asia	Bhutan	2016	11.5	13.8
	Indonesia	2016	9.3	6.3
	Myanmar	2016	8.7	6.5
	Thailand	2015	11.8	12.9
Western Pacific	Tonga	2017	12.4	14.0
	Vanuatu	2016	14.9	20.6
	Philippines	2015	10.2	14.9
	Brunei Darussalam	2014	9.5	6.6

### Participants' characteristics

Overall, 53.1% of participants identify as male whereas 46.9% as female. The majority were of age 15 or older (54.6%), in ASC classes (grades 7 to 9) (66.9%) and living in rural areas (51.2%). Of participants, 8.7% reported the lowest level of parental involvement. About 1 in 10 of the respondents indicated that they were always or, most of the time hungry due to not having food at home. The mental health parameters assessed indicated that 20.1 and 17.6% felt lonely and were so worried they could not sleep respectively. Approximately 39% of the participants reported that they were bullied during the prior month. At least 8% currently smoke cigarettes, 7% currently use marijuana, 8% ever used amphetamines or methamphetamines, and 15% reported ever using drugs. Table 2 delves into the demographics and characteristics of all participants.

**Table 2:** Demographics and characteristics of the students that participated in the GSHS Morocco 2016.

**Variables**	**Categories**	**N (%)**
Age	11 years old or younger	100 (1.5)
	12 years old	713 (10.7)
	13 years old	1,050 (15.8)
	14 years old	1,147 (17.3)
	15 years old	1,065 (16.1)
	16 years old	1,152 (17.4)
	17 years old	713 (10.7)
	18 years old or older	693 (10.4)
Sex	Male	3,488 (53.1)
	Female	3,085 (46.9)
School Grade	1 ASC (grade 7)	1,600 (24.4)
	2 ASC	1,322 (20.2)
	3 ASC	1,459 (22.3)
	Common Core	761 (11.6)
	1st year Bac	697 (10.6)
	2nd year Bac (grade 12)	706 (10.8)
Weight	Normal	4,761 (78.7)
	Underweight	507 (8.4)
	Overweight	625 (10.3)
	Obese	155 (2.6)
Location	Rural	3,452 (51.2)
	Urban	3,293 (48.8)
Hunger Frequency during the past 30 days	Never	4,288 (65.6)
	Rarely	706 (10.8)
	Sometimes	865 (13.2)
	Most of the time or always	680 (10.4)
Level of Parental Involvement	None	577 (8.7)
	Level 1	2,883 (43.5)
	Level 2	1,726 (26.1)
	Level 3	1,055 (15.9)
	Level 4	383 (5.8)
Most of the time or always felt lonely during the past 12 months	Most of the time or always	1,317 (20.1)
	No	5,240 (79.9)
Most of the time or always were so worried about something that they could not sleep at night during the past 12 months	Most of the time or always	1,171 (17.6)
	No	5,489 (82.4)
Were bullied during the last 30 days	Yes	2,466 (38.9)
	No	3,869 (61.1)
Currently smokes cigarettes	Yes	534 (8.3)
	No	5,913 (9.7)
Currently uses marijuana	Yes	453 (7.1)
	No	5,951 (92.9)
Ever used amphetamines or methamphetamines	Yes	464 (8.1)
	No	5,276 (91.9)
Used drugs before age 14 years	Yes	593 (71.3)
	No	239 (28.7)

### Bivariate and multivariate analysis

Suicidal ideation was found to be associated with multiple demographic variables, lack of parental involvement and risk behavior variables (*p* < 0.05) as indicated in Table 3. Bivariate associations revealed that adolescents who identified as male were less likely to express suicidal ideation as compared to females and an increase in hunger frequency increases the likelihood of suicidal ideation. Adolescents with increased parental involvement were associated with lower rates of suicidal ideation. Mental health and risky behavior parameters increased the likelihood of suicidal ideation. Multivariate analysis revealed an increase in suicidal ideation with increased age, identifying as female, lower school grade, lack of parental involvement and increased hunger frequency. In the multivariate analysis, the mental health and risky behavior parameters increased the likelihood of suicidal ideation: felt lonely most of the time (OR: 2.481; 95% CI: 2.091–2.944), so worried that they couldn't sleep (OR: 2.640; 95% CI: 2.220–3.141), bullied (OR: 2.145; 95% CI: 1.829–2.517), smoke cigarettes (OR: 3.081; 95% CI: 2.386–3.979), use marijuana (OR: 2.739; 95% CI: 2.058–3.647), and ever used amphetamines or methamphetamines (OR: 3.360; 95% CI: 2.552–4.423).

**Table 3:** Chi-squared and regression analysis of demographics and risk factors associated with suicidal ideation.

**Variable**	**Category**	**Suicidal ideation (%)**	**Bivariate association**		**Multivariate association**	
			**Unadjusted OR**	**95% CI**	**Adjusted OR**	**95% CI**
Age	11 years old or younger	9.2	0.495	0.233–1.052	0.192^*^	0.071–0.521
	12 years old	12.6	0.707^*^	0.523–0.957	0.397^*^	0.25–0.633
	13 years old	13.8	0.781	0.595–1.024	0.456^*^	0.301–0.689
	14 years old	15.8	0.919	0.708–1.192	0.610^*^	0.420–0.887
	15 years old	16.5	0.968	0.745–1.257	0.654^*^	0.457–0.937
	16 years old	17.6	1.042	0.808–1.344	0.730	0.525–1.017
	17 years old	18.8	1.135	0.86–1.498	1.030	0.751–1.413
	18 years old or older	17.0	1.000		1.000	
Sex	Male	15.0	0.868^*^	0.758–0.994	0.772^*^	0.661–0.903
	Female	16.9	1.000		1.000	
School Grade	1 ASC	15.5	1.145	0.884–1.484	2.607^*^	1.705–3.985
	2 ASC	14.5	1.055	0.807–1.378	1.966^*^	1.315–2.940
	3 ASC	17.5	1.324^*^	1.024–1.711	1.999^*^	1.393–2.868
	Common Core	15.4	1.138	0.848–1.526	1.578^*^	1.091–2.283
	1st year Bac	17.8	1.347^*^	1.005–1.806	1.53^*^	1.083–2.164
	2nd year Bac	13.8	1.000		1.000	
Weight	Normal	15.5	1.000		1.000	
	Underweight	12.6	0.789	0.596–1.044	0.777	0.578–1.044
	Overweight	17.7	1.178	0.942–1.473	1.179	0.930–1.494
	Obese	16.9	1.11	0.717–1.719	1.089	0.683–1.735
Location	Rural	15.9	0.975	0.853–1.114	0.936	0.802–1.093
	Urban	16.2	1.000		1.000	
Hunger Frequency during the past 30 days	Never	13.0	0.444^*^	0.362–0.544	0.478^*^	0.382–0.598
	Rarely	17.3	0.624^*^	0.477–0.816	0.654^*^	0.486–0.879
	Sometimes	22.3	0.854	0.668–1.092	0.796^*^	0.607–1.044
	Most of the time or always	25.1	1.000		1.000	
Level of Parental Involvement	None	12.6	1.000		1.000	
	Level 1	49.3	0.692^*^	0.555–0.864	0.731^*^	0.573–0.933
	Level 2	22.5	0.495^*^	0.388–0.631	0.507^*^	0.387–0.665
	Lever 3	12.1	0.412^*^	0.313–0.542	0.466^*^	0.344–0.631
	Level 4	3.5	0.313^*^	0.210–0.467	0.380^*^	0.246–0.587
Most of the time or always felt lonely during the past 12 months	Most of the time or always	29.1	2.894^*^	2.495–3.357	2.481^*^	2.091–2.944
	No	12.4	1.000		1.000	
Most of the time or always were so worried about something that they could not sleep at night during the past 12 months	Most of the time or always	30.2	2.908^*^	2.499–3.383	2.640^*^	2.220–3.141
	No	13.0	1.000		1.000	
Were bullied during the last 30 days	Yes	23.0	2.393^*^	2.079–2.755	2.145^*^	1.829–2.517
	No	11.1	1.000		1.000	
Currently smokes cigarettes	Yes	35.7	3.389^*^	2.736–4.199	3.081^*^	2.386–3.979
	No	14.1	1.000		1.000	
Currently uses marijuana	Yes	32.6	2.831^*^	2.227–3.600	2.739^*^	2.058–3.647
	No	14.6	1.000		1.000	
Ever used amphetamines or methamphetamines	Yes	36.6	3.657^*^	2.896–4.618	3.360^*^	2.552–4.423
	No	13.6	1.000		1.000	
Used drugs before age 14 years	Yes	35.0	1.299	0.919–1.836	0.827	0.513–1.334
	No	29.3	1.000		1.000	

* * p < 0.05*.

Additionally, suicidal planning was found to be associated with multiple demographic and risky behavior variables (*p* < 0.05) as indicated in Table 4. Bivariate analysis indicated that there is no statistically significant difference between male and female adolescents in terms of suicidal planning; however, multivariate analysis indicated that males are less likely to express suicidal planning (OR: 0.818; 95% CI: 0.696–0.961). Hunger frequency trends and parental involvement had similar results to that of suicidal planning in both bivariate and multivariate analysis. Multivariate analysis revealed multiple mental health and risky behavior parameters that increase the likelihood of suicidal planning (*p* < 0.05): felt lonely most of the time (OR:1.955; 95% CI: 1.632–2.342), so worried that they couldn't sleep (OR: 1.994; 95% CI: 1.657–2.399), bullied (OR: 1.715; 95% CI: 1.454–2.023), smoke cigarettes (OR: 2.920; 95% CI: 2.234–3.817), use marijuana (OR: 2.645; 95% CI: 1.971–3.549), and ever used amphetamines or methamphetamines (OR: 2.751; 95% CI: 2.070–3.656).

**Table 4:** Chi-squared and regression analysis of demographics and risk factors associated with suicidal planning.

**Variable**	**Category**	**Suicidal planning**	**Bivariate association**		**Multivariate association**	
			**Unadjusted OR**	**95% CI**	**Adjusted OR**	**95% CI**
Age	11 years old or younger	14.8	0.495	0.233–1.052	0.434^*^	0.190–0.990
	12 years old	12.8	0.707^*^	0.523–0.957	0.470^*^	0.288–0.765
	13 years old	14.8	0.781	0.595–1.024	0.569^*^	0.368–0.881
	14 years old	15.4	0.919	0.708–1.192	0.651^*^	0.434–0.979
	15 years old	15.0	0.968	0.745–1.257	0.707	0.476–1.049
	16 years old	15.5	1.042	0.808–1.344	0.846	0.584–1.227
	17 years old	14.2	1.135	0.86–1.498	1.095	0.766–1.566
	18 years old or older	12.0	1.000		1.000	
Sex	Male	14.6	1.003	0.871–1.155	0.818^*^	0.696–0.961
	Female	14.5	1.000		1.000	
School Grade	1 ASC	16.2	1.145	0.884–1.484	3.312^*^	2.107–5.208
	2 ASC	15.6	1.055	0.807–1.378	2.603^*^	1.689–4.010
	3 ASC	15.6	1.324^*^	1.024–1.711	2.162^*^	1.453–3.217
	Common Core	14.6	1.138	0.848–1.526	1.948^*^	1.304–2.909
	1st year Bac	10.5	1.347^*^	1.005–1.806	1.059	0.704–1.595
	2nd year Bac	10.0	1.000		1.000	
Weight	Normal	14.0	1.000		1.000	
	Underweight	14.9	1.071	0.821–1.397	0.997	0.750–1.325
	Overweight	15.0	1.082	0.853–1.373	1.132	0.883–1.450
	Obese	18.5	1.39	0.907–2.128	1.403	0.899–2.191
Location	Rural	15.7	1.204^*^	1.047–1.384	1.020	0.869–1.198
	Urban	13.4	1.000		1.000	
Hunger Frequency during the past 30 days	Never	12.2	0.444^*^	0.362–0.544	0.451^*^	0.360–0.565
	Rarely	14.3	0.624^*^	0.477–0.816	0.579^*^	0.426–0.788
	Sometimes	17.8	0.854	0.668−1.092	0.641^*^	0.484–0.849
	Most of the time or always	24.7	1.000		1.000	
Level of Parental Involvement	None	12.6	1.000		1.000	
	Level 1	48.6	0.701^*^	0.556–0.882	0.751^*^	0.581–0.969
	Level 2	23.9	0.545^*^	0.424–0.701	0.607^*^	0.459–0.803
	Lever 3	11.7	0.416^*^	0.312–0.554	0.496^*^	0.360–0.682
	Level 4	3.3	0.309^*^	0.202–0.473	0.409^*^	0.259–0.644
Most of the time or always felt lonely during the past 12 months	Most of the time or always	22.7	2.098^*^	1.792–2.457	1.955^*^	1.632–2.342
	No	12.3	1.000		1.000	
Most of the time or always were so worried about something that they could not sleep at night during the past 12 months	Most of the time or always	23.8	2.172^*^	1.847–2.553	1.994^*^	1.657–2.399
	No	12.6	1.000		1.000	
Were bullied during the last 30 days	Yes	19.2	1.926^*^	1.664–2.23	1.715^*^	1.454–2.023
	No	11.0	1.000		1.000	
Currently smokes cigarettes	Yes	29.9	2.975^*^	2.374–3.729	2.920^*^	2.234–3.817
	No	12.5	1.000		1.000	
Currently uses marijuana	Yes	33.8	3.447^*^	2.713–4.379	2.645^*^	1.971–3.549
	No	12.9	1.000		1.000	
Ever used amphetamines or methamphetamines	Yes	32.1	3.267^*^	2.567–4.158	2.751^*^	2.070–3.656
	No	12.6	1.000		1.000	
Used drugs before age 14 years	Yes	32.8	1.787^*^	1.224–2.609	1.261	0.740–2.149
	No	21.5	1.000		1.000	

* * p < 0.05*.

## Discussion

The study found that 1 in 6 and 1 in 7 school attending adolescents in Morocco reported suicide ideation and planning respectively. That, suicidality is positively associated with age, being a female, lack of parental support, increased hunger frequency, risky behaviors and worse mental health wellbeing. Studying psychosocial factors associated with completed suicide poses a significant challenge, alternatively, factors affecting ideation and planning can be assessed. It is challenging to compare the data to the global international trends due to sociocultural differences impacting the perception of suicide and thus affecting the willingness to self-report ideation and planning (1). However, the results and trends are comparable to those of other countries in the region with similar sociocultural influences (3, 21, 22).

The results indicate that the prevalence of suicidal ideation and planning among school-attending adolescents in Morocco is comparable to that of countries in the Eastern Mediterranean Region, which ranges from 16–21% and 9–17% respectively. In comparison to the other regions, GSHS data indicated that the prevalence of suicidal ideation and planning in Morocco is lower than that of countries in Africa and the Americans but higher than countries in South-East Asia and Western Pacific (Table 1). The results indicate that studying factors associated with suicide need to be socioculturally relevant to the said region as different factors may impact suicidality differently based on external factors.

Mental health and wellbeing in the Eastern Mediterranean Region are influenced by many parameters that are unique to the region. Belief and religiosity are found to be a source of wellbeing and a protective factor against suicide (23). The region contains an amalgam of religions: Islam, Christianity, and Judaism. Islam accounts for the belief of 90% of the citizens in the region (24). Religion plays a significant role in the lives of individuals in the region. Considering Islam, Christianity, and Judaism prohibit suicide, it may in turn affect the prevalence of suicide. The scarcity of data and research assessing suicide in Muslim-majority countries further challenges understanding suicide and associated factors in the region (4). Additionally, studies have shown the potential presence of a relationship between climate change and mental health (25). As such, the similarities in the climate in the region may play a role in the trends of mental illnesses particularly anxiety and depression (24).

The Eastern Mediterranean Region is rich in culture and historic backgrounds. Culturally, families in the region are more closely knit than other regions (24). Being in a supportive, healthy family-oriented environment is found to be protective of mental illness (26). Both cultural background and a family environment supports mental wellbeing (24).

The common misconceptions and negative attitudes toward mental illness generate stigma that impacts access to mental healthcare and mental wellbeing. Due to the influence of culture and religion in the Arab world, mental illness is often viewed as the result of a higher power. For instance, Muslims often view mental illness as the “evil eye” or “jinn possession,” Christians often view it as the “devil possession” and in certain countries due to cultural influences it is viewed as a “case of contamination” that can be accidentally contracted by “stepping on sorcery or drinking it” as seen in Morocco (27).

The results indicate that the prevalence of suicidal ideation and planning are similar in the region, which could be due to the sociocultural differences as highlighted above. Additionally, the results support the global sex differences in suicidal ideation, which indicates that females are more likely to express suicidal ideation (28).

Additionally, the demographic data associated with suicidal ideation was similar to that of other countries in the region. For instance, an increasing hunger frequency was associated with an increase in suicidal ideation (22). Multiple studies exploring variables that increase the likelihood of suicidal ideation also revealed an increased likelihood associated with increased bullying, cigarette smoking, feeling lonely, feeling worried, drug use, and marijuana use (15, 22, 28). Interestingly however, our study revealed an association between suicidal ideation and increasing age, which was similar to a study conducted in Lebanon using data from 2005 but opposing another study conducted in Lebanon in 2020 (15, 22).

Suicidal planning trends and associations were similar to that of other countries in the region. A study assessing factors associated with suicidal ideation and planning amongst Palestinian adolescents also revealed that feeling lonely, feeling worried, experiencing bullying, smoking cigarettes, using marijuana, and using amphetamines increased the likelihood of suicidal planning. Similarly, our study revealed similar findings using both bivariate and multivariate analysis. Additionally, our results revealed that drug use before the age of 14 increased the likelihood of suicidal planning. In terms of participants characteristics, our data indicates an association between increasing hunger frequency, living in a rural area and suicidal planning. Both parameters may be associated with socioeconomic status affecting food scarcity. Additionally, it was found that decreasing educational levels was associated with increasing rates of suicidal planning.

The study adds to the limited data available in the region. Based on the results and regional trends, national systemic interventions need to be studied to decrease the prevalence of the growing public health threat. Interventions need to be aimed at tackling hunger frequency, creating supportive school environments, introducing school counselors in order to recognize early signs and intervene early, and educate students about mental health and the consequences of cigarette smoking, marijuana use, and drug use. Additionally, significant efforts are needed to address the stigma associated with mental illness, which impacts individuals' willingness to seek help.

It is challenging to study the factors associated with completed suicide, as such suicidal ideation and planning are used instead to draw inferences. Additionally, it is also challenging to conduct a prospective study to explore factors associated with completed suicide. Alternatively, a cross-sectional design was used. The main limitation of such study design is reverse causality, and thus only allows us to determine associations rather than causation. Additional limitations include under-reporting, sample size not including adolescents not enrolled in schools, and the lack of pertinent demographics such as socioeconomic status, religion, and family dynamics. On the other hand, the study's large sample size ensures the diversity and representativeness of the data, and since the data was a part of the WHO's Global Schools Health Survey it allows for better comparison between regions and countries. Further research needs to be conducted to consider specific sociocultural differences in order to better understand their impact on suicidality.

Based on the study results, monitoring of adolescent mental health wellbeing in schools should be a priority, awareness campaigns with parents should be initiated and culturally acceptable interventions should be developed and tested to address suicidality. The effort should come from all involved parties (Government, School, Parents and Adolescents) focusing on psychoeducation, taking into account sociocultural factors and also tackling the associated stigma to build community-based interventions.

## Data availability statement

The original contributions presented in the study are included in the article/supplementary material, further inquiries can be directed to the corresponding author.

## Ethics statement

The studies involving human participants were reviewed and approved by Moroccan Ministry of Health. Written informed consent from the participants' legal guardian/next of kin was not required to participate in this study in accordance with the national legislation and the institutional requirements.

## Author contributions

AT conducted the literature review, statistical analysis, and manuscript writing under the supervision and mentorship of ZM. Both authors contributed to the article and approved the submitted version.

## Funding

This paper uses data from the Global Youth Tobacco Survey (GYTS). GYTS is supported by the World Health Organization and the US Centers for Disease Control and Prevention.

## Conflict of interest

The authors declare that the research was conducted in the absence of any commercial or financial relationships that could be construed as a potential conflict of interest.

## Publisher's note

All claims expressed in this article are solely those of the authors and do not necessarily represent those of their affiliated organizations, or those of the publisher, the editors and the reviewers. Any product that may be evaluated in this article, or claim that may be made by its manufacturer, is not guaranteed or endorsed by the publisher.
